# Advances in understanding ischemic acute kidney injury

**DOI:** 10.1186/1741-7015-9-11

**Published:** 2011-02-02

**Authors:** Raj Munshi, Christine Hsu, Jonathan Himmelfarb

**Affiliations:** 1 Department of Pediatrics, Division of Nephrology, University of Washington and Seattle Children's Hospital, Seattle, WA, USA; 2 Kidney Research Institute, Department of Medicine, Division of Nephrology, University of Washington, Seattle, WA, USA

## Abstract

Acute kidney injury (AKI) is independently associated with increased morbidity and mortality. Ischemia is the leading cause of AKI, and short of supportive measures, no currently available therapy can effectively treat or prevent ischemic AKI. This paper discusses recent developments in the understanding of ischemic AKI pathophysiology, the emerging relationship between ischemic AKI and development of progressive chronic kidney disease, and promising novel therapies currently under investigation. On the basis of recent breakthroughs in understanding the pathophysiology of ischemic AKI, therapies that can treat or even prevent ischemic AKI may become a reality in the near future.

## Introduction

Acute kidney injury (AKI) is independently associated with increased morbidity and mortality. AKI has been associated with increased length of hospital stay and adjusted odds ratios of 4.1 for hospital mortality and 2.0 for discharge to short- and long-term care facilities [1]. Among critically ill patients, period prevalence of AKI in a multinational, multicenter study comprising approximately 30,000 patients was demonstrated to be 5.7%. The major contributing factors were shock (septic shock 47.5%), major surgery and hypovolemia. Observed mortality in this study was 60.3% compared with the predicted mortality of 45.6% using the simplified acute physiology score II [2]. Short of supportive measures, no available therapy has definitively proven to effectively treat or prevent ischemic AKI. Recent experimental research has helped elucidate the pathophysiologic basis behind ischemic AKI, and therapies that can treat or even prevent ischemic AKI may become a reality in the near future. This paper discusses recent developments in the understanding of ischemic AKI pathophysiology, the emerging relationship between ischemic AKI and the development of progressive chronic kidney disease (CKD), and promising novel therapies currently under investigation.

## Pathobiology of ischemia

**Figure 1:**
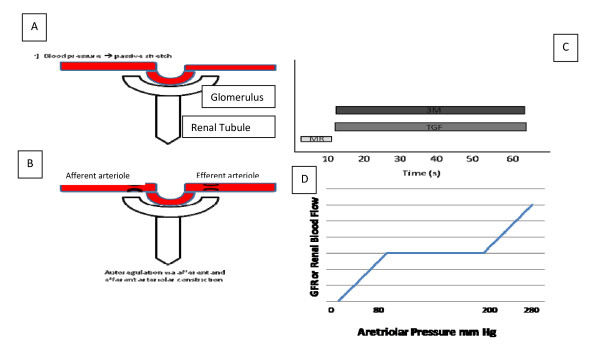
**Renal vascular autoregulation involves three mechanisms: the myogenic reflex (MR), tubuloglomerular feedback (TGF) and a recently discovered third regulatory mechanism (3M)**. MR refers to the contraction of the smooth muscle in response to stretching forces. TGF is a kidney-specific regulatory mechanism that causes vasoconstriction of the afferent arterioles in response to increased luminal concentration of chloride at the macula densa in the early distal tubule. 3 M takes place chronologically after MR and TGF and was discovered when it was observed that renovascular autoregulation took place well after MR took place and after TGF was inhibited by furosemide administration. **(A) **Increase in pressure leads to passive stretch. **(B) **Regulatory mechanisms are activated to maintain renal blood flow via smooth muscle contraction of afferent and efferent arterioles. **(C) **Three regulatory mechanisms work in concert. MR occurs early and is completed in 10 seconds. TGF and 3 M are late responses, with a delay of 10 to 15 seconds, and take 30 to 60 seconds to complete. **(D) **Autoregulation maintains constant renal blood flow and glomerular flow rate while systemic blood pressure fluctuates between 80 and 180 mmHg.

While the human adult kidneys account for 2% of total body weight, they receive approximately 25% of the cardiac output. This facilitates the high rates of glomerular filtration required for the precise regulation of the body's fluid and electrolyte balance. With autoregulation, glomerular filtration rate (GFR) is tightly maintained despite changes in arteriolar pressure; GFR remains constant while systemic blood pressure fluctuates between 80 and 180 mmHg (Figure 1) [3]. Under physiologic conditions, the renal cortex, which contains the majority of the glomeruli (or filtering units), receives most of the renal blood flow, whereas the medulla receives approximately 10% [4]. During ischemia, the reduction in blood flow is regional rather than uniform throughout the kidney. The decrease in renal blood flow is more prominent in the outer medulla than in the cortex [5] . The source of medullary blood flow arises from the efferent arterioles of the juxtamedullary glomeruli giving rise to the vasa recta. This serially organized renal microvasculature allows for the countercurrent mechanisms vital for sodium balance. Tubular transport in the medullary thick ascending limb (TAL) and S3 segment of the proximal tubule demands high oxygen consumption. If the blood supply becomes interrupted transiently, the oxygen balance is maintained by reducing GFR and solute transport to the TAL. This protective mechanism is undermined by production of reactive oxygen species that further decrease medullary blood flow and increase TAL activity. Clinically, this translates to increased injury during the reperfusion phase, when the oxygen balance is tilted toward consumption because of increased demand for tubular transport. As discussed below, the initial injury further initiates a cascade of events leading to endothelial damage. An inflammatory response also leads to vascular congestion that propagates the hypoxic environment and reduces the ability to clear the toxic radicals. Thus the corticomedullary region is the most vulnerable region of the kidney to tubular injury, inflammation and vascular alterations that extend the cellular injury beyond the initial insult and propagate continued hypoperfusion [6].

Observed vascular alterations include disruption of the endothelial actin cytoskeleton, leading to the detachment of cells from the endothelial monolayer.

**Figure 2:**
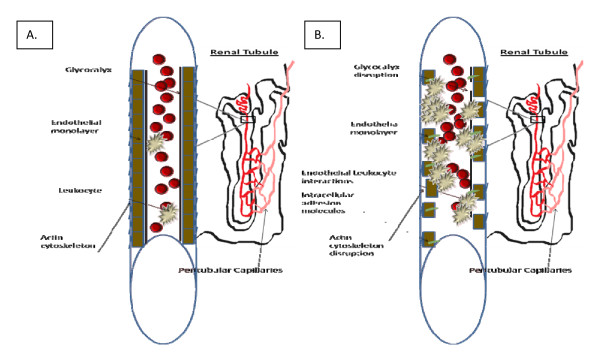
**Peritubular capillary schematic under two conditions**. **(A) **Cross-section of normal peritubular capillaries with an intact endothelial monolayer anchored by the actin cytoskeleton and covered with the glycocalyx. **(B) **Endothelial alterations due to acute kidney injury, such as glycocalyx disruption, actin cytoskeleton disruption, detachment of the endothelial monolayer with increased endothelium-leukocyte interaction and expression of intracellular adhesion molecules.

This results in altered endothelial barrier function, vascular reactivity and increased permeability [6,7]. The inflammatory response leads to enhanced leukocyte-endothelium interactions, leading to increased expression of intercellular adhesion molecules (ICAM) such as ICAM-1, P- and E-selectin and B7-1. Experiments that ablate or decrease the expression of these molecules attenuate kidney injury and preserve kidney function in animal models of ischemic and septic AKI [6]. The leukocyte-endothelium interaction also leads to shedding of the sulfated glycosaminoglycan-rich layer, or glycocalyx, of the endothelium. This subsequently initiates downstream signaling cascades and increases access of leukocytes to transmigrate the endothelium into the injured kidney [8]. These factors collectively create an environment that leads to further tubular injury and propagates the initial insult, resulting in a further decrease in GFR and kidney function (Figure 2).

## Progression to chronic kidney disease

Clinical episodes of AKI may have lasting implications, and new data suggest that these include increasing the likelihood of subsequent development of CKD [9-12]. The chronic hypoxia hypothesis was formulated by Fine *et al. *[13] more than a decade ago on the basis of renal biopsy findings in patients with CKD that demonstrated marked rarefaction of the peritubular capillaries [12]. Their hypothesis proposed that primary glomerular injury leads to reduced postglomerular flow, which culminates in peritubular capillary loss [13]. This creates a hypoxic environment that produces a fibrotic response that further propagates injury by affecting adjacent unaffected capillaries. Another perspective is that renal injury triggers an inflammatory response that recruits profibrotic cytokines such as transforming growth factor 1 and further induces the transformation of renal epithelial and endothelial cells to myofibroblasts, a process called epithelial mesenchymal transition [14]. This leads to fibroblast and myofibroblast production in the matrix, with subsequent tubulointerstitial injury and atrophy [11]. The histopathological hallmark of CKD is tubulointerstitial fibrosis, and the degree of fibrosis is the best predictor for the progression to end-stage renal disease [10].

In addition to profibrotic processes, hypoxia also suppresses matrix degradation via reduced expression and activity of matrix metalloproteinases such as collagen metalloproteinase-I [15]. Fibrosis itself is not sufficient to impede the function of the kidney. The eventual loss of the microvasculature creates a hypoxic milieu and produces the progressive nature of fibrosis. Rarefaction of peritubular capillaries was observed during detailed examination of biopsies from patients with CKD [16] and in animal models [17] . In the animal models, there was a direct relationship between peritubular capillary rarefaction and the development of glomerular and tubulointerstitial scarring. Further studies in rats showed that there was a permanent reduction in peritubular capillary density after recovery from ischemic AKI, suggesting that the acute ischemic insult leads to a chronic hypoxic state [18].

Studies using pimonidazole, which binds to hypoxic cells *in vivo*, demonstrated hypoxia of the kidney in association with reduction in the peritubular capillary blood flow at an early stage of a model of progressive glomerulonephritis induced by uninephrectomy and repeated injection of antimesangial Thy1 antibody [19]. Pimonidazole staining also showed the existence of renal hypoxia in polycystic kidney disease and diabetic nephropathy. Hypoxia has also been observed in puromycin-induced nephritic syndrome, aging kidney and the remnant kidney model in rats. These studies demonstrated that the reduction in the density of peritubular capillaries leads to a hypoxic environment and suggest a mechanism for the observed progression from AKI to CKD to a final common pathway to end-stage kidney disease [20].

## Hypoxia-inducible factor

A hypoxic environment increases the transcription of genes involved in angiogenesis, erythropoiesis and anaerobic energy metabolism. Hypoxia-inducible factor (HIF) is thought to play a major role in this process [21]. HIF is a basic helix-loop-helix transcription factor composed of α- and β-subunits. In normoxia, the α-subunit is degraded by proteasomal degradation via proline hydroxylation by HIF-specific prolylhydroxylases (PHDs), which leads to binding of von Hippel-Lindau protein and targeted degradation through the ubiquitin-proteosome pathway. In hypoxic conditions, the HIF-1α subunit escapes degradation, translocates to the nucleus, forms a heterodimer with HIF-1β and binds to the hypoxia-responsive element motif. This leads to increased activation of between 100 and 200 genes involved in angiogenesis, erythropoiesis and energy metabolism [22].

Currently, there is an intense research focus on the stabilization of HIF as a novel therapeutic option for renal hypoxic injury, including ischemic reperfusion injury and CKD. In an animal model of renal mass reduction, Song *et al. *[23] showed that activation of HIF by dimethyloxalylglycine attenuated the increase in proteinuria and structural damage by preventing podocyte injury. The renoprotection was accompanied by a reduction of oxidative stress, inflammation and fibrosis. Several other experimental studies demonstrated protection against ischemia-reperfusion (I/R) injury by stabilization of HIF through carbon monoxide, cobalt chloride administration, xenon anesthesia and PHD inhibitors (PHD-I) [19,24,25]. Bernhardt *et al. *[26] showed improved short- and long-term outcomes in rats that underwent allogenic kidney transplant and were pretreated with PHD-I. Human studies have also been performed using PHD-I as a novel treatment for anemia through the induction of erythropoietin expression [27].

There are conflicting data regarding whether HIF promotes fibrosis and progression to CKD. Higgins *et al. *[28,29] demonstrated in *HIF-1α*-knockout mice that stabilization of HIF-1 leads to increased transcription of the profibrotic gene connective tissue growth factor (*Ctgf*).

They further demonstrated enhanced epithelial-to-mesenchymal transition *in vitro *by HIF-1α and induced epithelial cell migration through upregulation of lysyl oxidase genes in mice subjected to unilateral ureteral obstruction. HIF also has a central role in tumor stabilization.

Angiogenesis is essential for tumor survival. As cells grow and divide, the neoplastic compartment rapidly expands past the diffusion distance of oxygen in tissue. As regions of the tumor become hypoxic, HIF mediates a cellular response resulting in angiogenesis and anaerobic energy metabolism. In renal cell carcinoma, there is a biallelic inactivation of the E3 ubiquitin ligase responsible for targeting the HIFα subunit for degradation. HIF-2α seems to have a greater role in renal cell carcinoma in disease progression while HIF-1α has a greater role in solid tumors, demonstrating that their specificity is important for specific tumor propagation and survival. The role of HIF in tumor propagation must be seriously considered, and tumorigenesis must be closely monitored, with regard to the development of PHD-I [30].

## Ischemia reperfusion injury and preconditioning

As previously noted, ischemia is a leading cause of AKI. Ischemia and subsequent reperfusion elicit AKI through endothelial dysfunction, leukocyte-mediated inflammation and decreased microvascular blood flow [31]. In a rat model of renal I/R injury, upregulation of proteins related to kidney development occurred in a specific spatial and temporal sequence. The initial insult was associated with increased expression of injury and/or apoptosis markers, followed by a regeneration phase with upregulation of kidney mesenchymal proteins followed by tubular markers, with endothelial marker expression occurring throughout early and midregeneration [32]. This provides insights into the mechanisms behind ischemic AKI and recovery and suggests specific therapeutic targets that may be effective only during a narrow window around an ischemic insult.

In a mouse model of I/R injury, sphingosine-1-phosphate (S1PR) agonists attenuated AKI indirectly by redirecting lymphocytes away from the kidney [33,34] and directly by acting on the proximal tubule [35]. The selective S1PR agonist SEW2871 reduced apoptosis in cultured mouse proximal tubule epithelial cells induced by I/R injury. Deletion of Dicer, a key enzyme for microRNA production, was also associated with resistance to ischemic AKI in mice [36].

Although I/R injury elicits AKI, episodes of nonlethal ischemia may protect the kidneys against future ischemic AKI. Ischemic preconditioning (IPC) refers to eliciting nonlethal ischemia to provide protection against a future ischemic insult. In mice, a first episode of renal ischemia protected against AKI after a second episode of renal ischemia [37]. Such renal protection was found to be mediated by Tregulatory (Treg) lymphocytes, which inhibit neutrophil and macrophage accumulation in the kidney, tubular necrosis and AKI [38]. Treg cell depletion reversed this protection, and Treg cell infusion mimicked the effect of IPC. Other pathways through which IPC confers protection include upregulation of cell survival pathways and downregulation of apoptotic pathways [37,39].

Remote ischemic preconditioning (RIPC) refers to eliciting nonlethal ischemia to an organ to protect a distant organ from future injury. In adults undergoing abdominal aortic aneurysm repair, RIPC was induced by two cycles of intermittent cross-clamping of the common iliac artery. This was associated with a 23% decrease in AKI (30% vs. 7%, *P *= 0.009), which was defined by a rise in serum creatinine to >177 μM/L (2 mg/dL) [40]. However, in another study, in patients undergoing multivessel coronary artery bypass graft (CABG) surgery, the incidence of AKI in those who received RIPC was no different than that of controls [41]. In another study, RIPC was elicited with three 5-minute cycles of upper-extremity ischemia elicited using a blood pressure cuff [42]. In the most recently published retrospective study of nondiabetic adults undergoing CABG surgery, RIPC was also elicited with three 5-minute cycles of upper-extremity ischemia using a blood pressure cuff. Compared with controls, fewer patients receiving RIPC developed AKI as defined using the Acute Kidney Injury Network definition [43]. RIPC has been demonstrated to attenuate systemic inflammation [43,44]. Further study of IPC and RIPC in kidney protection may not only elucidate the pathobiology of ischemic AKI but also lead to effective AKI treatments if indeed IPC and RIPC are renoprotective.

## Conclusion

Pathophysiologic changes secondary to ischemic AKI lead to endothelial injury with disruption of the endothelial monolayer, as well as increased leukocyte endothelial interaction and recruitment. The inflammatory process eventually leads to rarefaction of the peritubular capillaries, shifting the fragile balance of oxygen supply and demand to the corticomedullary junction toward a negative oxygen balance. The shift in balance causes a hypoxic environment, leading to accumulation of fibrosis and development of CKI. Factors involved in adaptation to this hypoxic environment, such as HIF, may have potential as therapeutic targets, but their application must be balanced against the profibrotic and tumor-producing potential of HIF stabilizers. Moreover, further evaluation of IPC and RIPC may not only elucidate the pathobiology of ischemic AKI but also hold promise as effective therapies in primary AKI prevention.

## Competing interests

The authors declare that they have no competing interests.

## Authors' contributions

RM drafted the manuscript and created the figures. CH drafted the manuscript. JH developed an outline for and helped draft the manuscript. All authors read and approved the final manuscript.

## Pre-publication history

The pre-publication history for this paper can be accessed here:

http://www.biomedcentral.com/1741-7015/9/11/prepub
